# Improving HPV Vaccine Coverage in Tennessee: Addressing Barriers and Expanding Access for Mid-Adults

**DOI:** 10.3390/pathogens14040311

**Published:** 2025-03-25

**Authors:** Donald J. Alcendor, Patricia Matthews-Juarez, Mohammad Tabatabai, Derek Wilus, James E. K. Hildreth, Paul D. Juarez

**Affiliations:** 1 Center for AIDS Health Disparities Research, Department of Microbiology, Immunology, and Physiology, School of Medicine, Meharry Medical College, 1005 Dr. D.B. Todd Jr. Blvd., Nashville, TN 37208-3599, USA; 2 Department of Family & Community Medicine, Meharry Medical College, 1005 D.B. Todd Jr. Blvd., Nashville, TN 37208-3501, USA; 3 Department of Public Health, School of Global Health, Meharry Medical College, 1005 Dr. D.B. Todd Jr. Blvd., Nashville, TN 37208-3501, USA

**Keywords:** human papilloma virus, HPV, Tennessee, vaccine, vaccine hesitancy, vaccination rate, mid-adults

## Abstract

Human papillomavirus (HPV) is the most common sexually transmitted infection in the US and the world. Infection with high-risk oncogenic HPV strains has been shown to induce cellular transformation leading to anogenital and oropharyngeal cancers. The HPV vaccine, first developed in 2006 for females aged 9–26 years, has been demonstrated to be safe and effective in preventing 90% of all HPV-associated cancers. However, vaccine hesitancy, misinformation, and barriers to vaccine access has resulted in suboptimal vaccination rates among adolescent populations, especially in rural communities in the South. HPV vaccine coverage in Tennessee is currently below the national average and below the Healthy People 2030 goal of an 80% vaccination rate for individuals 13–17 years old based on recommendation guidelines for up-to-date HPV vaccination status as of 2022. HPV vaccination rates for Tennesseans with private insurance in 2022 were 68% and 38% for those that were uninsured. Up-to-date HPV vaccination rates in 2022 for Tennesseans were 58% and 46% for those living in urban communities and rural communities, respectively. Overall, HPV-associated cancers rates are higher in Tennessee, at 12.9/100,000 compared to the overall rate in the US of 11.8/100,000 persons in 2022. Interventions to improve HPV vaccine awareness, education, and access could improve vaccine confidence and uptake, especially among rural and uninsured populations in Tennessee. Most recently, the Advisory Committee on Immunization Practices (ACIP) expanded recommendations for HPV vaccinations for some individuals aged 27–45 years who were not vaccinated at a younger age, with shared clinical decision making. Further research is needed to evaluate the impact of this recommendation on HPV vaccination rates and cancer prevention in Tennessee.

## 1. Introduction

Human papillomavirus (HPV) is the most commonly sexually transmitted infection in the US and globally. There are more than 150 strains of HPV, including high-risk strains that can induce cellular transformation that can lead to cancer [1]. The HPV virion was first observed by Strauss et al. in 1949 [2] via electron microscopy, but it was Herald Zur Hausen in 1976 that proposed HPV as the etiological cause of cervical cancer, leading to the isolation of HPV 16 and 18 from cancerous cervical tissue [3]. HPV is readily transmitted via sex and skin-to-skin contact, with the virus gaining access to mucosal surfaces via micro-abrasions in the cervical epithelium basement membrane leading to virus uptake by endocytosis and subsequent initiation of nuclear replication [4]. HPV is widespread, with over 80% of sexually active individuals contracting it at some point in their lives. Spontaneous viral clearance usually occurs within two years after infection; however, persistent infections with HPV can lead to benign lesions such as genital warts as well malignancies of the cervix, penis, anus, vagina, vulva, and oropharynx [5]. Individuals with HIV/AIDS or who are iatrogenically immunosuppressed are less likely to achieve viral clearance and are susceptible to HPV-associated disease and HPV-associated malignancies [6]. HPV vaccine hesitancy remains a significant challenge in Tennessee and the Southern US. The Tennessee HPV vaccination rate for 13–17-year-olds as of 2022 was 74% compared to the national average of 76%. [7]. Although vaccine coverage of greater than or equal to one dose (1</=) has increased in Tennessee by 19% since 2016, and UTD (up to date) coverage has increased by 28%, HPV vaccine coverage in Tennessee is currently below the national average and below the Healthy People 2030 goal of 80% UTD [7]. UTD vaccination rates among Tennesseans 13–17 years of age is lowest, at 38% among uninsured teens compared to 58% of teens with Medicaid insurance and 52% of teens with private insurance [7]. Even more, rates of cervical and oropharyngeal cancer, which are the most common HPV-associated cancers, were higher in Tennessee, at 5.7 and 6.6/100,000 persons, respectively, with the US at 5.0 and 6.5/100,000 persons, respectively.

However oropharyngeal squamous cell carcinoma has surpassed the incidence of cervical cancer for the past 15 years. Vaccine education, awareness, and access could improve uptake among medically underserved populations in Tennessee, especially in rural communities and among uninsured populations, which would move Tennessee closer to the Healthy People 2030 goal of 80% UTD coverage. Overall, HPV-associated cancer rates are higher in Tennessee, at 12.9/100,000 compared to the overall rate in the US of 11.8/100,000 persons [7]. Most recently, the ACIP committee expanded recommendation for HPV vaccinations for some individuals 27–45 years who were not vaccinated at a younger age, with shared clinical decision making [8]. Here, we examine the ACIP recommendation for Tennesseans and its potential impact on the medically underserved. We also present a brief overview of HPV biology and associated cancers, the HPV vaccine and vaccination rates in the US, HPV disease burden and vaccination rates in Tennessee, HPV vaccination rates by race/ethnicity, insurance coverage, and urbanicity in Tennessee, HPV vaccination rates for individuals 27–45 years old (mid-adults) and the implications for mid-adults in Tennessee, disparities in HPV vaccine health literacy, and strategies that could be implemented to improve HPV vaccine confidence and uptake in Tennessee.

## 2. Methods

County-wide HPV vaccination data for the year 2023 were obtained from the Tennessee Department of Health’s Immunization Statistics. These data measure the HPV vaccination rate for the West, Middle, and East Tennessee regions.

Descriptive, exploratory, and graphical data analyses were performed on HPV vaccination rates for 11–17 and 18–26 age groups by region and county. Differences in the means of the HPV vaccination rate were compared between the two age groups using the independent t-test. For each age group, a one-way ANOVA was performed to examine the differences in the vaccination rates among the three Tennessee regions. Pairwise comparisons were made using Bonferroni post hoc test. In addition to the regional analysis, a county-wide comparative analysis was performed.

## 3. Results

### 3.1. Human Papilloma Virus and Associated Cancers

**Figure 1:**
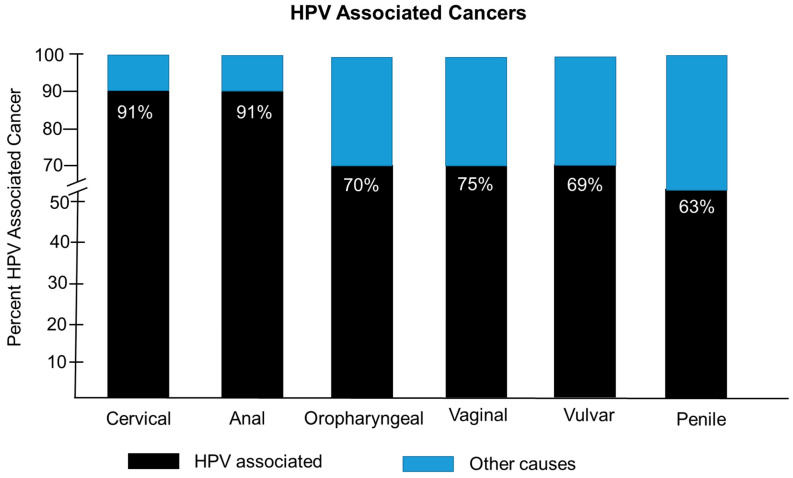
The percentages of HPV-associated cancers. The percentages of HPV-associated cancers shown as black bars and other causes shown by blue bars, including cervical, anal, oropharyngeal, vaginal, vulvar, and penile cancer.

(HPV) is a member of the Papillomaviridae family of small non-enveloped viruses with a double-stranded circular DNA genome [9]. Infections can be persistent and are usually self-limiting. HPV represents a large family of viruses that are divided into five different genera, namely the Alpha, Beta, Gamma, Mu, and Nu papillomaviruses [10]. The Alpha, Mu, and Nu papillomaviruses are clinically associated with benign lesions in the form of warts, while the Beta and Gamma genera are associated cutaneous asymptomatic infections. The Alpha genera HPVs are also known to cause infections of oral and anogenital mucosal epithelial cells, and a subset of these HPVs have oncogenic potential, leading to cancers. The HPV genome is 7–8 kb in length and encodes eight viral proteins that include six early proteins, E1, E2, E4, E5, E6, and E7, which are required for genome replication and protection against host immune defenses [9]. There are two additional late proteins, L1 and L2, which encode the viral capsid proteins that facilitate genome packing that are designated as important for viral entry and initiation of infection in permissive cells [10]. The L2 protein is essential in facilitating L1 assembly into viral-like particles (VLPs) and viral genome encapsidation [11]. Upon viral entry, the E6 and E7 oncoproteins target tumor suppressor proteins p53 and Rb, respectively, for suppression and dysfunction [12]. Integration of high-risk HPV genomes into permissive cells can lead to over-expression of the E6/E7 protein, which can drive tumorigenesis. Infections with low-risk HPVs rarely result in genomic integration, often resulting in benign low-grade lesions [13]. HPV is known to be associated with six different types of cancer in humans. HPV has been shown to be associated >91% of cervical cancers, 91% of anal cancers, 70% of oropharyngeal cancers, 75% of vaginal cancers, 69% of vulvar cancers, and 63% of penile cancers (Figure 1).

**Figure 2:**
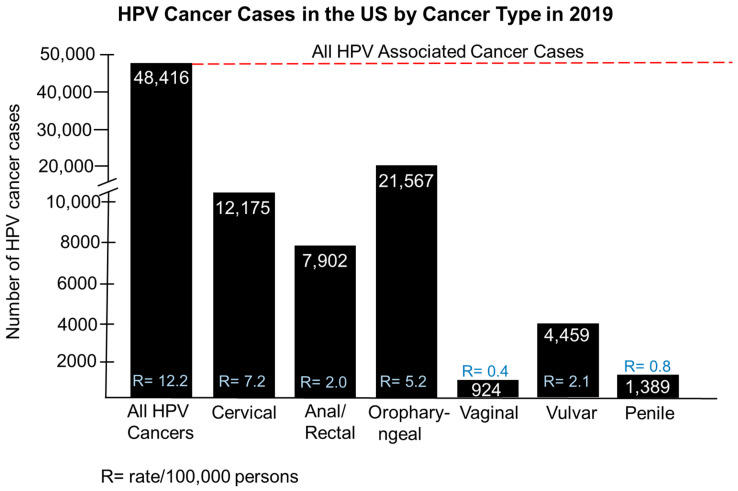
HPV cancer cases in the US by cancer types in 2019. The black bars represent all HPV cancers (represented by the red dotted line) as well as the respective case number breakdown by cancer type. R = cancer rate, which is the number of cancer cases/100,000 persons.

In 2019, there were 48,416 HPV-associated cancer cases in the US or 12.2 cases per 100,000 persons. Among these cases, there were 12,175 cases of cervical cancer (7.2/100,000), 7902 cases of colorectal cancer (2.0 cases/100,000), 21,567 cases of oropharyngeal cancer (5.2/100,000), 824 cases of vaginal cancer (0.4/100,000), 4459 cases of vulvar cancer (0.4/100,000), and 1389 cases of penile cancer (0.8/100,000) (Figure 2).

### 3.2. HPV Vaccine and Vaccination Rates in the US

**Figure 3:**
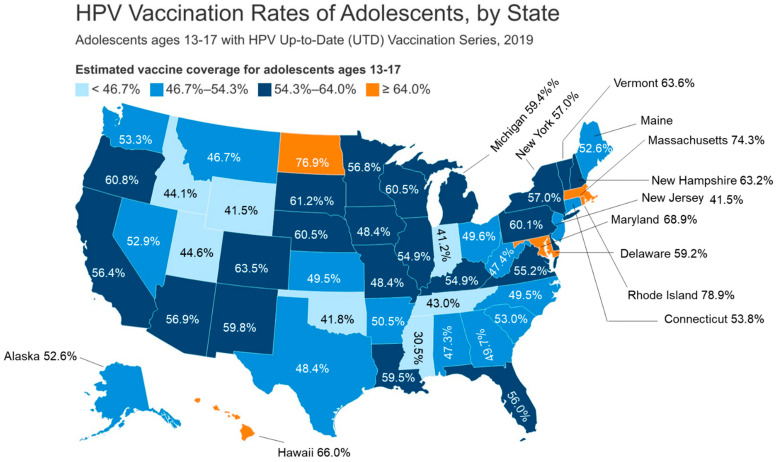
HPV vaccination rates in the US for adolescents, by state, for 2019. The numbers for each state represent estimated coverage of adolescents aged 13–17 years that had received an HPV Up-To-Date (UTD) vaccination series. State colors represent HPV vaccination levels for the different states in the US, with light blue representing UTD vaccination rates <46.7% and orange showing UDT HPV vaccination rates >/= 64.0%.

There are three preventive HPV vaccines licensed for use in the US, including Cervarix, a bivalent vaccine or 2vHPV (GlaxoSmithKline), Gardasil (Merck), a quadrivalent vaccine or 4vHPV, and most recently, the 9valent/9vHPV vaccine known as Gardasil 9 (Merck). Most recently, two additional bivalent (HPV 16 and 18) HPV vaccines have been approved in China for HPV-associated cervical cancers [14,15]. The greatest HPV cancer burden is caused by HPV 16 and 18, and these two types are targeted by all three HPV vaccines (2vHPV, 4vHPV, and 9vHPV) licensed in the US; however, v4HPV and 9vHPV protect against HPV types 6 and 11, which cause anogenital warts. Most notably, v9HPV also protects against five other high-risk types, which include HPV 31, 33, 45, 52, and 58 [16]. However, since 2016, Gardasil 9 is the only HPV vaccine available in the US, which protects against nine different HPV types and six different human cancers [17]. All three HPV vaccines employ recombinant DNA technology, which involves the purified L1 capsid protein undergoing self-assembly to form a virion shell or virus-like particles, or VLPs, as the antigen. The most recent FDA-approved HPV vaccine, the 9vHPV vaccine, is a noninfectious virus-like particle vaccine (VLP), first approved by the FDA in December of 2014, for use in females aged 9 through 26 years and males aged 9 through 15 years. Safety and efficacy data for v9HPV to date has not been determined for children under 9 years of age. Upon review of clinical trial data for 4vHPV in women aged 24 to 45 years old along with the bridging of safety and immunogenic profiles observed in women and men for 4vHPV, the FDA in 2018 expanded the age range for 9vHPV from 9 through 26 years old to 9 through 45 years old for both men and women [16]. In 2019, the ACIP then approved shared clinical decision making for mid-adults that lack vaccine protection and were at risk of novel HPV type acquisition. HPV vaccination rates vary in the US by region, and Southern states consistently have some of the lowest vaccination rates in the country (Figure 3) [17]. Vaccination rates for 2019 among adolescents 13–17 years old with the up-to-date vaccination series are shown in Figure 3. Only the states Rhode Island (78.9%), North Dakota (76.9%), Massachusetts (74.3%), Maryland (68.9%), and Hawaii (66.0%) had vaccination rates >/= 64% (shown in orange) (Figure 3) [18]. Only seven states had vaccination rates > or = to 46.7%, which included Utah (44.6%), Idaho (44.1%), Tennessee (43.0%), Oklahoma (41.89%), Wyoming (41.5%), and Indiana (41.2%), with the lowest HPV vaccination rate found in Mississippi, at 30.5% (Figure 3) [18]. The HPV vaccine is required for girls to enter the sixth grade in Virginia and Washington DC. However, parents can opt out for medical, religious, or moral reasons. However, all students entering the seventh grade in Rhode Island must be vaccinated for HPV. Laws that govern mandatory HPV vaccinations in the US have been controversial because of the concern that HPV vaccination could encourage sexual activity among teens. However, studies have refuted these concerns and have shown that HPV vaccine initiation is not associated with unwarranted sexual activity among teens [19].

### 3.3. HPV Disease Burden and Vaccinations in Tennessee

Tennessee vaccination rates consistently fall short of public health goals outlined by the Healthy People 2030 Initiative of an 80% vaccination rate for individuals aged 13–17 years [20]. Vaccination rates for HPV in Tennessee are consistently in the bottom fifth of states in the US [20]. There is a high incidence of cervical cancer and cervical cancer mortality in Tennessee, which warrants increasing HPV vaccination coverage in Tennessee, which could prevent more than 90% of these cancers. The two most common HPV-associated cancers in Tennessee are oropharyngeal cancer and cervical cancer (Table 1). In 2022, the oropharyngeal cancer rate in Tennessee was higher than the national average of 5.0%/100,000, at 5.7%/100,000. The cervical cancer rate in 2022 in Tennessee was 6.6%/100,000 compared to 6.5%/100,000 in the US overall (Table 1). Overall, Tennessee has a higher cancer rate for all HPV-associated cancers, at 12.9%/100,000 compared to a national average of 11.8%/100,000 (Table 1) [21]. There are also gender differences observed with oropharyngeal cancers in Tennessee compared to the national average (Table 1). In Tennessee, males account for 9.9%/100,000 of oropharyngeal cancers compared to 1.9%/100,000 for females, compared to a US average of 8.8%/100,000 for males and 1.6%/100,000 for females (Table 1) [21]. The improvement of vaccine uptake is essential for reducing the incidence of HPV-associated cancers in Tennessee. Currently, the HPV vaccine is not recommended for school attendance in Tennessee; only four jurisdictions in the US require HPV vaccinations for school attendance, including Hawaii, Virginia, Rhode Island, and the District of Columbia (DC) [22]. In April of 2023, laws were passed in Tennessee that prohibit primary care providers from vaccinating anyone under 18 years old without informed consent from a parent or legal guardian [23]. The law requires that medical providers maintain vaccination records on all children, and failure to comply could result in their license being revoked [23]. The current HPV vaccination recommendations based on age, dose, and dose interval are outlined in Table 2 [24]. The HPV vaccine is recommended for males and females ages 9 to 45 years old (Table 2). Ages 9 to 14 years old require a two doses that are 6 to 12 months apart. Ages 15 to 45 years require a three-dose regimen. Ages 15 to 26 years old require a second dose that should be given 1–2 months after the first and a third dose 1–6 months after the first dose (Table 2). Individuals aged 27 to 45 years old should consult with their primary care physician (shared clinical decision making) to determine if they would benefit from HPV vaccinations (Table 2) [24].

**Figure 4:**
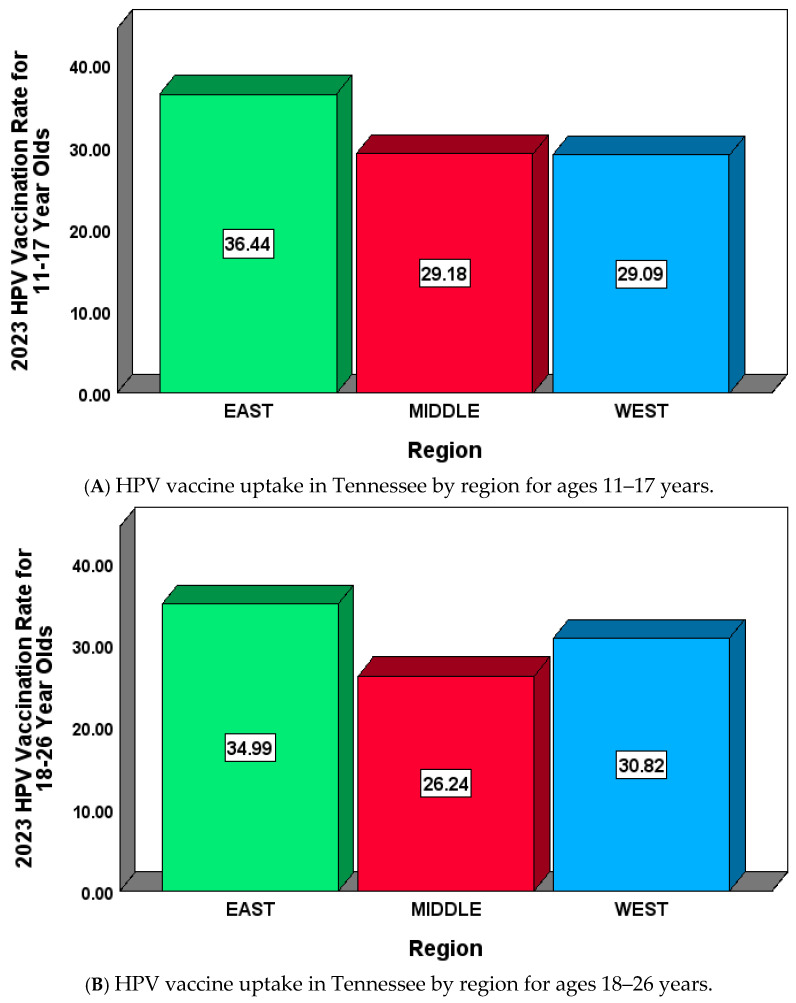
HPV vaccination rates in Tennessee by region in the year 2023 for ages 11–17 (**A**) and 18–26 (**B**). Mean vaccination rate values are shown inside the colored bars for East, Middle, and West Tennessee regions.

We examined HPV vaccination rates in Tennessee by region in the year 2023 for ages 11–17 and 18–26 (Figure 4A,B) [25,26]. An analysis of regional vaccination rates for 11–17-year-olds shows that the East region of Tennessee had a mean HPV vaccination rate of 36.44% [SD = 7.01, 95% CI = (33.95, 38.92)] (Figure 4A). The minimum HPV vaccination rate for the East region was 19.80%, which belonged to Claiborne County, and the maximum HPV vaccination rate was 52.00%, which belonged to Cocke County. The Middle Tennessee region had a mean HPV vaccination rate of 29.18% [SD = 9.26, 95% CI = (26.26, 32.11)] (Figure 4A). The lowest HPV vaccination rate for Middle Tennessee belonged to Lewis County (13.00%), and the highest rate was 51.30%, which belonged to Davidson County. The West Tennessee region had a mean HPV vaccination rate of 29.09% [SD = 7.13, 95% CI = (25.84, 32.33)]. Decatur County had the lowest HPV vaccination rate in the West region (18.00%), while Lake County had the highest rate of 49.6%. For the 11–17-year-old age group, there was a significant difference among the three Tennessee regions with respect to mean HPV vaccination rates (*p*-value < 0.001) (Figure 4A). The Bonferroni pairwise comparison revealed a significant difference in the mean HPV vaccination rate between West and East regions (*p*-value = 0.005). In addition, there was a significant difference in the mean HPV vaccination rate for 11–17-year-olds between Middle Tennessee and East Tennessee regions (*p*-value < 0.001).

Similarly, the regional analysis for 18–26-year-olds for East Tennessee [26] had a mean HPV vaccination rate of 34.99% [SD = 7.24, 95% CI = (32.42, 37.56)] (Figure 4B). Claiborne County had the lowest HPV vaccination rate (21.00%), and Meigs County had the highest rate, at 52.7%. In the Middle Tennessee region, the mean HPV vaccination rate for 18–26-year-olds was 26.24% [SD = 6.29, 95% CI = (24.26, 28.22)] (Figure 4B). Moore County in Middle Tennessee had the lowest HPV vaccination rate of 15.1% for 18–26-year-olds, and the maximum rate of 44.1% belonged to Van Buren. For 18–26-year-olds, the mean HPV vaccination rate for the West Tennessee region was 30.82% [SD = 7.37, 95% CI = (27.47, 34.18)]. Chester County in West Tennessee had the lowest HPV vaccination rate, at 15%, while Benton County had the highest HPV vaccination rate, at 44.7%. For the 18–26-year-old age group, there was a significant regional difference in the mean HPV vaccination rate (*p*-value < 0.001) (Figure 4B). A pairwise comparison indicated a significant difference between the mean HPV vaccination rates of Middle Tennessee and East Tennessee (*p*-value < 0.001). There was also a significant difference in the HPV rates of West Tennessee and Middle Tennessee for the 18–26 age group (*p*-value = 0.044).

Figure 4A illustrates the East, Middle, and West regional mean HPV vaccination rates for 2023 for the 11–17 age group. The highest mean HPV vaccination rate belonged to the East Tennessee region (36.44%), and the lowest mean rate was from the Middle Tennessee region (29.18%). Similarly, for the age group 18–26, the East Tennessee region maintained the highest mean HPV vaccination rate (34.99%), while the Middle Tennessee region had the lowest rate (26.24%), as shown in (Figure 4B).

**Figure 5:**
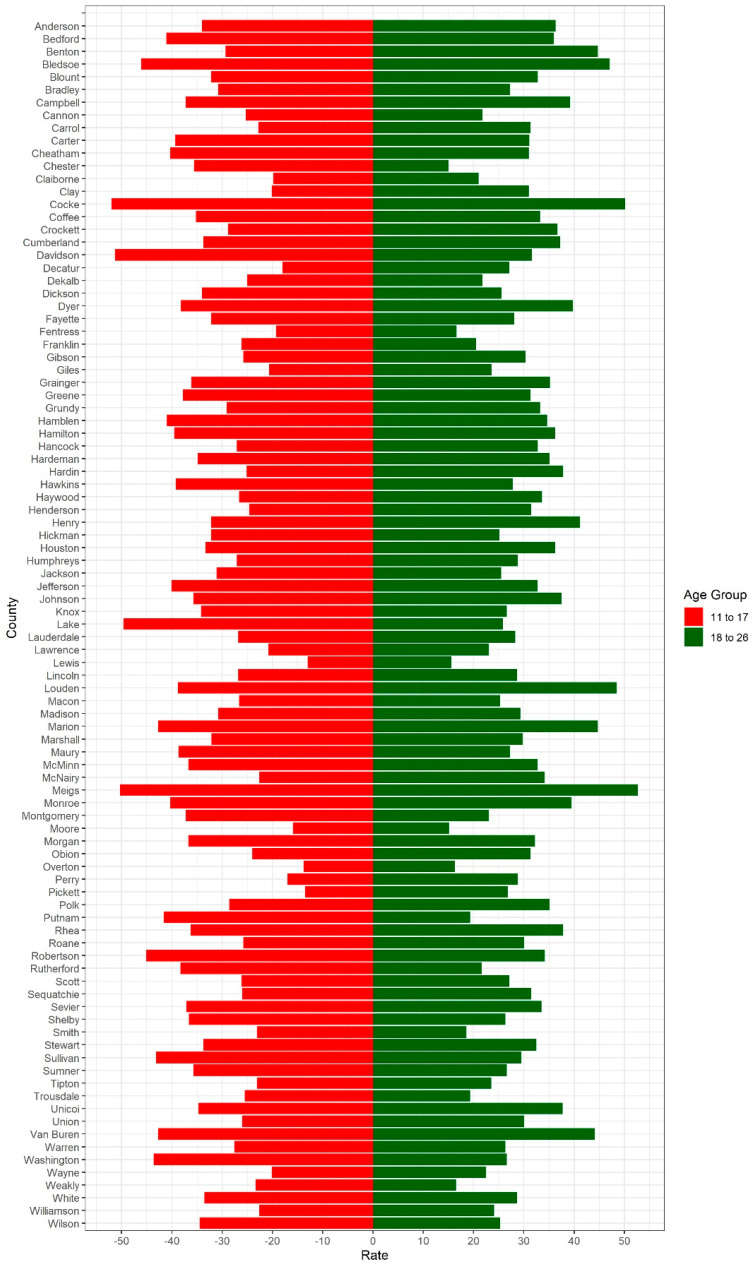
HPV vaccination rates for ages 11–17 and 18–26 in Tennessee by county as reported by TennIIs in 2023. County HPV vaccination rates in 2023 are stratified by age group.

We also examined HPV vaccination rates for ages 11–17 and 18–26 in Tennessee by county as reported by TennIIs in 2023 (Figure 5). Among 11–17-year-olds, Lewis County had the lowest HPV vaccination rate, at 13%, and Cocke County had the highest rate, at 52%. Among 18–26-year-olds, Chester County had the lowest HPV vaccination rate, at 15%, while Meigs County had the highest rate, at 52.7%. There was no significant difference between the mean HPV vaccination rate between 11–17- and 18–26-year-olds (*p*-value = 0.249). In 2023, the mean HPV vaccination rate for 11–17-year-olds in Tennessee was 31.68%, with a standard deviation of 8.73%. Similarly, the mean HPV vaccination rate in 2023 for 18–26-year-olds in Tennessee was 30.29%, with a standard deviation of 7.82% (Figure 5).

### 3.4. HPV Vaccinaation Rates by Race/Ethnicity, Insurance Coverage, and Urbanicity in Tennessee

**Figure 6:**
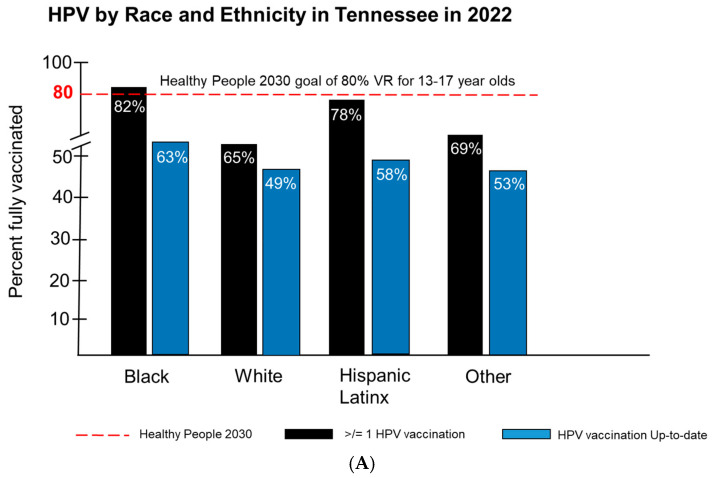

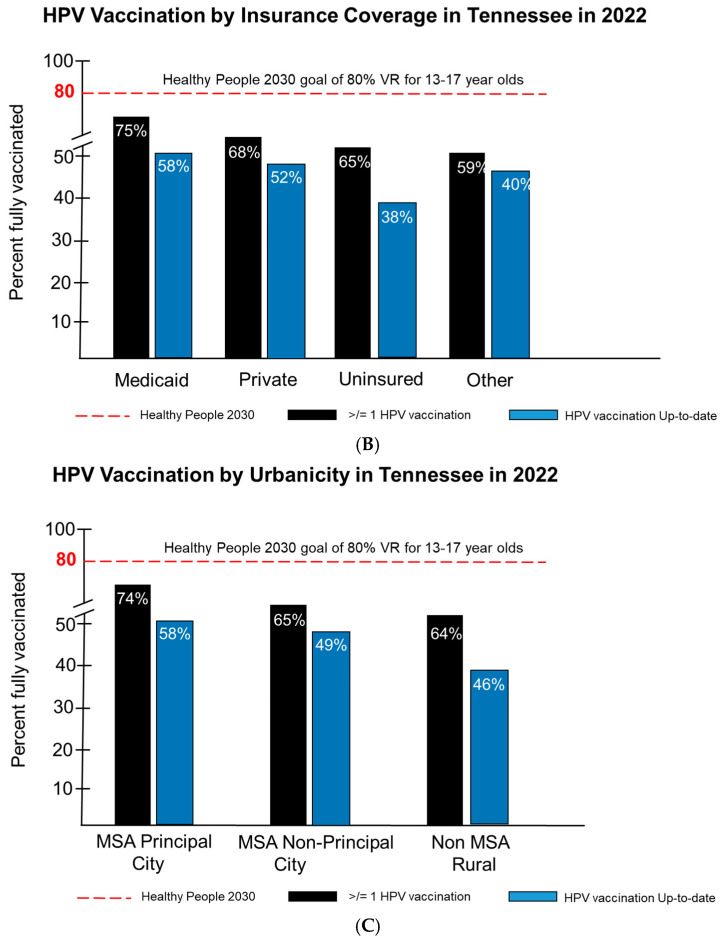
HPV vaccination rates by race/ethnicity, insurance coverage, and urbanicity in Tennessee in 2022. HPV vaccination rate by race and ethnicity for 13 to 17-year-olds in 2022 (**A**). HPV vaccination rates in Tennessee based on insurance coverage for 13 to 17-year-olds in 2022 (**B**). HPV vaccination rates in Tennessee based on urbanicity for 13 to 17-year-olds in 2022 (**C**). All rates were compared to the Healthy People 2030 goal of an 80% vaccination (shown by the red dotted line) rate for 13–17-year-olds in the US that received >/= to 1 dose of the HPV vaccine and those that completed the dosing regimen and were up to date (UTD). Rates are shown for Black, White, Hispanic/Latinx, and Other categories.

We examined the 2022HPV vaccination rates for Tennessee by race/ethnicity, insurance coverage, and urbanicity for 13–17-year-olds (Figure 6A). The Healthy People 2030 goal is an 80% vaccination rate for 13–17-year-olds in the US with regard to receiving >/= to one dose of the HPV vaccine or completing the dosing regimen and being up to date (UTD) [26]. Tennessee fell short of these goals, with the exception of Black Tennesseans, who achieved an HPV vaccination rate of 82% (Figure 6A) compared to White (65%), Hispanic/Latinx (58%), and others (69%) for populations receiving </= to one dose [27]. For populations of 13–17 years that were to be up to date, no race or ethnic group in Tennessee reached a rate of 80%. The rates observed for all races and ethnicities included Black Tennesseans at 63%, followed by Hispanic/Latinx populations at 58%, others at 53%, and White Tennesseans at 49% (Figure 6A) [27]. Analysis of HPV vaccination rates based on insurance coverage showed that a high level of vaccine initiation was observed among 13–17-year-olds receiving >/= 1 dose compared to those that were UTD for their HPV vaccinations (Figure 6B). Those individuals insured by Medicaid had the highest level (75%) of vaccine initiation for all racial and ethnic groups receiving >/= 1 dose, followed by private insurance, the uninsured, and other forms of insurance (Figure 6B). Among 13–17-year-olds that were UTD for their HPV vaccinations, populations insured by Medicaid had the highest UTD vaccination levels, at 58%, followed by private insurance (52%), other (40%), and the uninsured, with the lowest UTD vaccination rate of 38% (Figure 6B). None of the insured or uninsured populations reached the Healthy People 2030 goal of an 80% vaccination rate for either incomplete or UTD HPV vaccinations [27]. Finally, we examined the 2022 HPV vaccination rates in Tennessee for 13–17-year-olds. No racial or ethnic group in Tennessee reached the Healthy People 2030 goal of an 80% vaccination rate for those receiving >/= 1 dose or for those that were UTD for their HPV vaccinations (Figure 6C). Populations living in MSA Principal City locations had the highest level of vaccine initiation (>/=1 dose), at 74%, compared to those living in non-MSA Principal City locations (65%), and the vaccination initiation rate observed among those populations living in non-MSA rural communities was the lowest, at 64% (Figure 6C) [27].

### 3.5. HPV Vaccination Rates for Individuals 27–45 Years Old (Mid-Adults)

In 2018, the FDA approved HPV vaccinations for individuals 27–45 years old (mid-adults) as supported by the Advisory Committee on Immunization Practices (ACIP) recommendations of shared clinical decision making rather than the routine vaccination of mid-adults [28]. This decision by the ACIP is based on individuals who are not adequately vaccinated and could be at risk for new HPV infections and might benefit from HPV vaccinations as mid-adults. Expanding vaccination efforts to this group could significantly reduce disease burden. FDA approval of the HPV vaccine for individuals 27–47 years was based on reported safety and efficacy data. A study by Munoz et al. demonstrated 90.5% efficacy in reducing HPV infections in women 24 to 45 years old [29]. In a HPV vaccine clinical trial study by Castellsagué et al., the authors observed efficacies of 88.7% and 79.9% protection against cervical intraepithelial neoplasia (CIN) and external genital lesions in women 24–45 years old [30]. However, the HPV vaccine has been shown to be most effective in individuals with no prior exposure to HPV [31,32]. There have been very few studies performed on HPV uptake among mid-adults in the US.

However, a cross-sectional study performed by Kasting et al., employing data from the 2017 National Health Interview Survey, showed that up to 47.2% of 27 to 45-year-olds had initiated vaccination at age 19 or older [33,34,35]. In addition, the survey showed that participants with higher education such as a bachelor’s degree or higher, having insurance, and of female gender were more likely to receive HPV vaccinations as mid-adults. ACIP’s shared clinical decision-making model requires healthcare provider consultations, which could enhance HPV education and awareness. This strategy would also allow timely diagnosis and treatment of HPV-associated clinical disease as well as support up-to-date HPV vaccinations. However, medically underserved populations who are uninsured or underinsured and may not have a primary care provider are more likely to be less educated about HPV and HPV-associated diseases and less likely to initiate HPV vaccination. Studies by Reiter et al. show that recommendations by medical providers and the perceived importance of preventing HPV-associated disease was a significant factor in initiating HPV vaccination in a study with women 18 to 45 years old [36]. Even more, a study by Weldon et al. in mid-adults showed that 80% of participants wanted more information about the vaccine prior to decision making, and 87.8% reported that medical providers were the primary source of information about the HPV vaccine [37].

**Figure 7:**
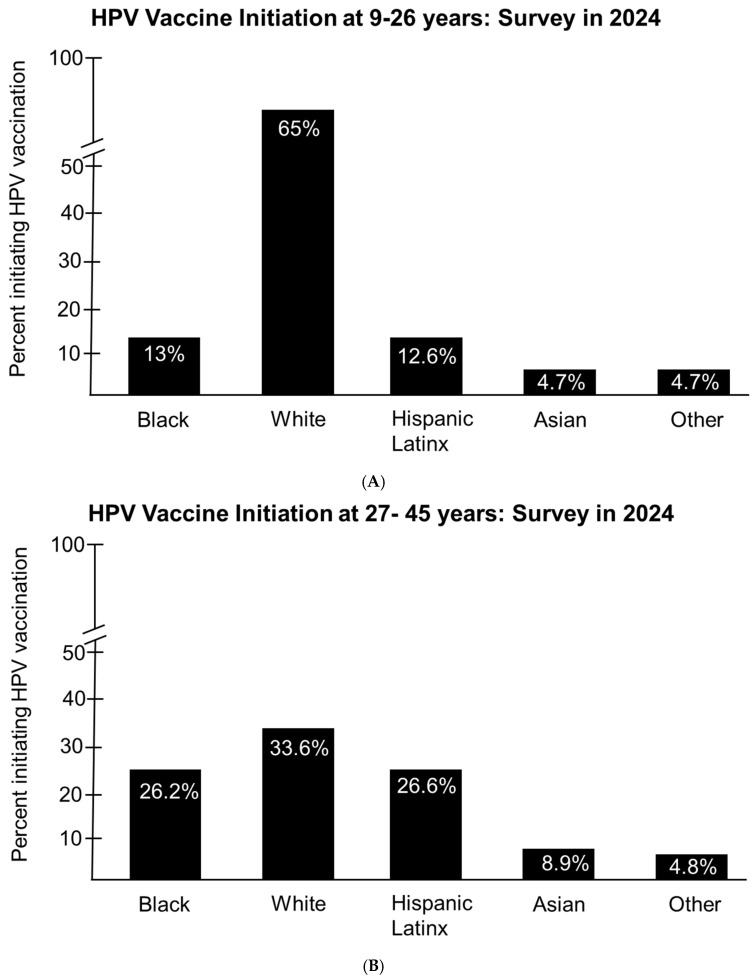
Vaccine initiation rates for ages 9–26 and 27–45 based on data from the 2019 National Health Interview Survey and reported in 2024. Shown are the percentages of HPV vaccine initiation for ages 9–26 years in black bars for Black, White, Hispanic/Latinx, and Other categories (**A**). The percentages of HPV vaccine initiation for ages 27–45 years in black bars for Black, White, Hispanic/Latinx, and Other categories (**B**).

A recent study by Akpan et al. that employed a national sample of 27–45-year-olds using the Andersen’s Behavioral Model of Health Services Use showed that persons of female gender, persons designated as being from certain racial/ethnic groups, and persons with higher education attainment were more likely to initiate HPV vaccinations for this age group [38]. In addition, the study showed that participants in relationships, those identifying as non-Hispanic Asian persons, and Hispanic/Latinx participants were less likely to have ever received the HPV vaccine as mid-adults [38]. They also found that persons that identified as female in gender as well as Hispanic, non-Hispanic Black, and non-Hispanic Asian race/ethnicity were more likely to initiate HPV vaccination after age 26 [38]. The study by Akpan et al. employed the analysis of data from the 2019 National Health Interview Survey. The study involved 8556 individuals classified as mid-adults aged 27–45 and a separate group of 7307 mid-adults that self-reported to have been vaccinated for HPV along with individuals who were unvaccinated for HPV. The overall outcomes of the study were HPV vaccination and vaccination initiation. Independent variables were aligned with Andersen’s Behavioral Model of Health Services Use. The study compared HPV vaccine initiation reported among individuals 9–26 and 27–45 years. Vaccination initiation among individuals aged 9–26 years was highest among White participants, at 65%, with 13% and 12.6% among Hispanic/Latinx and Black participants, respectively (Figure 7A). Asian and Other participants had the lowest level of vaccine initiation, with both at 4.7% (Figure 7A). They also examined HPV vaccine initiation among mid-adults aged 27–45 and found a significant difference between vaccine initiation among White participants, from 65% for those aged 9–26 years to 33.6% for those aged 27–45 years (Figure 7B) [38]. However, they observed a significant increase in HPV vaccine initiation among Blacks and Hispanic/Latinx participants from 13% and 12.6% for those aged 9–26 years to 26.2% and 26.6% when compared to mid-adults, respectively (Figure 7B). They also observed a significant increase in HPV vaccine initiation among Asian participants, from 4.7% to 8.9%, when they compared 9–26-year-olds to mid-adults; however, they found no significant increase in participants identified as Other (Figure 7B) [38].

The collection of data for HPV vaccination initiation for individuals 27–45 years (mid-adults) to our knowledge has not been reported for Tennessee. HPV vaccination rates among adolescents are suboptimal, which suggests that there is a pool of unvaccinated adults that are eligible and could benefit from vaccination [39]. HPV vaccinations would benefit mid-adults that develop new sexual partnerships, with potential exposure to sexually transmitted infections that would increase their risk of acquiring new strains of HPV [40]. Lewis et al. reported that 15–35% of sexually active women and 23–33% of sexually active men 25 to 49 years old in the US are infected with at least one or more high-risk HPV types [41]. These findings suggest that there is ongoing transmission of high-risk HPV types among mid-adults, which would warrant HPV vaccination in this at-risk population. Even more, the humoral response to HPV infection was found to be more robust when employing the 9vHPV vaccine compared to natural immunity to protect against reinfection [42,43]. As reviewed by the ACIP committee in their recommendation for the HPV vaccination of mid-adults, multiple models demonstrate a significant cost savings and reduction in disease burden when implementing 9vHPV vaccinations for all eligible mid-adults [44,45,46,47]. Regardless of exposure to an infection with ≥1 HPV type over their life-course, few mid-adults have immunity to all HPV types covered by the 9vHPV vaccine [41]. Even more, physicians will also need training regarding shared clinical decision making when recommending the HPV vaccine to mid-adults. A study by Hurley et al. showed that 42% of primary care providers recommended the HPV vaccine for their mid-adult patients. In the same study, 57% of providers did not know what information to share with their mid-adult patients [48]. However, mid-adult populations that are medically underserved and are less likely to engage primary care physicians for their health needs may not have an opportunity to participate in shared clinical decision making to facilitate HPV vaccine initiation.

The cost of and interest in initiation and uptake of the HPV vaccine among mid-adults will likely require further study to show a public health benefit as a rationale for data collection in this population, a topic that remains controversial. The associated cost will have to be justified by states in the US that currently do not collect HPV vaccine uptake data in this population. In Tennessee, this will require an aggressive health initiative to achieve policy changes that provide additional funding to Tennessee Departments of Health, supported by public and private insurance. Increasing HPV vaccination rates for children aged 9–11 years, young teens, and adolescents before sexual debut in Tennessee could greatly reduce the number of mid-adults requiring HPV vaccinations and would greatly reduce HPV-associated disease burden in this population.

### 3.6. Disparities in HPV Vaccine Health Literacy

Studies have shown disparities in HPV-associated cancers, which disproportionately affect medically underserved populations [49,50,51,52]. Disparities in HPV vaccine health literacy can contribute to disparities in vaccine access and uptake. Racial and ethnic disparities are associated with HPV vaccine access and uptake. Recent studies have shown that HPV health literacy is significantly lower among Black and other racial/ethnic minorities regarding HPV awareness, the vaccine, and HPV-associated malignancies [53]. Studies by Adjei et al. identified disparities in HPV vaccine awareness and uptake among adolescents and young adults [54]. In the study, Black populations were 33% (95% CI: 0.47– and 44% (95% CI: 0.39–0.81) less likely than non-Hispanic Whites to have heard of HPV and the HPV vaccine, respectively. Hispanics were 27% (95% CI: 0.52–1.02) and 53% (95% CI: 0.34–0.64) less likely than non-Hispanic Whites to have heard of HPV and the HPV vaccine, respectively [54]. In a study by King et al., gaps in knowledge regarding HPV vaccinations in mid-adult populations were observed in both patients and providers [55]. It is essential to develop interventions to improve HPV vaccine health literacy in medically underserved communities.

### 3.7. Strategies That Could Be Implemented to Improve HPV Vaccine Confidence and Uptake in Tennessee

In Tennessee, there are 95 counties, and 78 of these counties are designated as rural counties. These rural counties have less health care infrastructure, have fewer primary care physicians, have poor Social Determinant of Health (SDOH) levels, exhibit less educational attainment and lower health literacy, are distrustful of the government and the medical establishment, are highly exposed to misinformation about vaccines, and are more likely to be vaccine hesitant when compared to urban communities in Tennessee [56,57,58]. Misinformation about vaccine safety, combined with logistical barriers in rural communities, contributes to low HPV vaccine uptake. Improving health literacy among populations who have less educational attainment, especially in rural communities in Tennessee, could reduce vaccine hesitancy and improve HPV vaccine confidence and uptake [59]. In general, HPV vaccine coverage in rural communities is low, and the incidence of HPV-associated disease burden and cancer remains high [60]. Transportation to engage medical providers may represent an access barrier for rural communities in Tennessee. The deployment of mobile medical units staffed with physicians, nurses, and community health care workers could support the shared clinical decision making recommended by ACIP for HPV vaccinations among mid-adults in Tennessee [61]. Health fairs, town halls, faith-based community events, wellness visits by medical providers, and telehealth education programs can expand HPV vaccine access and related services to medically underserved communities in Tennessee. A longitudinal analysis of vaccination trends and barriers in Tennessee could provide insights that over time could result in policy changes that positively impact HPV vaccine uptake. Expanding Medicaid coverage in Tennessee and the broader Southern US could increase healthcare access, including HPV vaccinations, for uninsured populations. HPV vaccination outreach and interventions should not be focused on females only, suggesting that HPV affects only females, but should include males to achieve equity in reducing HPV infection and associated disease burden in the general population. HPV should be designated as a gender-neutral vaccination due to known HPV-induced malignancies in males (penile, anal, oropharyngeal cancers). In addition, gender-neutral vaccination (GNV) has been endorsed globally and implemented not only to battle the forthcoming oropharyngeal cancer epidemic, but also to address suboptimal vaccination rates in girls, predominantly to prevent HPV16 infections.

## 4. Conclusions

Each year in the US, approximately 48,000 HPV-associated cancers occur, and approximately 80% of these cancers could have been prevented with an HPV vaccination [62]. HPV-associated cancer rates in Tennessee are consistently higher than the national average. During the period 1999 to 2015, cervical cancer rates decreased in the US by 1.6% per year, and oropharyngeal cancer incidence rates increased 2.7% per year among men and 0.8% per year among women [63]. Specific interventions to reduce HPV vaccine hesitancy and improve vaccine initiation and completion of the HPV dosing series are essential for Tennessee to achieve the Healthy People 2030 goal of an 80% vaccination rate for respective age groups. HPV vaccine uptake in Tennessee has shown to be greatly influenced by age, race, insurance status, and urbanicity. Strategies to achieve HPV vaccine equity should improve HPV vaccine initiation in Tennessee to achieve the Healthy People 2030 goals. Even more, HPV vaccinations for mid-adults are currently not being captured in Tennessee, to our current knowledge; ACIP recommendations of shared clinical decision making will be required to support vaccine initiation and completion of HPV vaccine dosing in this population. Addressing disparities in vaccine literacy and reducing hesitancy in particular in rural and underserved communities must be prioritized to achieve public health goals.

## Figures and Tables

**Table 1:** Cancer rates for the two most common HPV-related cancers in Tennessee compared to the national average and by gender.

Two Most Common HPV Related Cancer in Tennessee			
	All HPV Cancers	Oropharyngeal Cancer	Cervical Cancer
United States Overall	11.8%	5.0%	6.5%
Tennessee Overall	12.9%	5.7%	6.6%

**Gender Differences**			
United States Overall	Male 10.9% Female 12.9%	Male 8.8% Female 1.6%	TN is ranked in the Top 30 nationally in cervical Cancer rates
Tennessee Overall	Male 11.9% Female 14.0%	Male 9.9% Female 1.9%	
Incidence rates shown in cases per 100,000			

**Table 2:** Current HPV vaccination recommendation by the CDC for males and females ages 9–45 years.

HPV Vaccination Recommendation				
	**On Time**	**Late**	**Extra Dose**	**Consultation**
**Age**	9–12 years	13–14 years	15–26 years	27–45 years
**Doses #**	2 Doses	2 Doses	3 Doses	3 Doses
**Dosing interval**	Each dose 6–12 months apart	Each dose 6–12 months apart	Second Dose 1–2 months after first; Third dose 1–6 months after first dose	Consult with your primary care physician to determine if they recommend an HPV vaccination

## Data Availability

This manuscript did not report any laboratory-based data.
